# The combination approach of SVM and ECOC for powerful identification and classification of transcription factor

**DOI:** 10.1186/1471-2105-9-282

**Published:** 2008-06-16

**Authors:** Guangyong Zheng, Ziliang Qian, Qing Yang, Chaochun Wei, Lu Xie, Yangyong Zhu, Yixue Li

**Affiliations:** 1 School of Life Sciences, Fudan University, 220 Handan Road, Shanghai 200433, PR China; 2 Department of Computing and Information Technology, Fudan University, 220 Handan Road, Shanghai 200433, PR China; 3 Bioinformatics Center, Key Lab of Systems Biology, Shanghai Institutes for Biological Sciences, Chinese Academy of Sciences, 320 Yueyang Road, Shanghai 200031, PR China; 4 College of Life Sciences and Technology, Shanghai Jiaotong University, 800 Dongchuan Road, Shanghai 200240, PR China; 5 Shanghai Center for Bioinformation Technology, 100 Qinzhou Road, Shanghai 200235, PR China; 6 Graduate School of the Chinese Academy of Sciences, 19 Yuquan Road, Beijing 100039, PR China

## Abstract

**Background:**

Transcription factors (TFs) are core functional proteins which play important roles in gene expression control, and they are key factors for gene regulation network construction. Traditionally, they were identified and classified through experimental approaches. In order to save time and reduce costs, many computational methods have been developed to identify TFs from new proteins and to classify the resulted TFs. Though these methods have facilitated screening of TFs to some extent, low accuracy is still a common problem. With the fast growing number of new proteins, more precise algorithms for identifying TFs from new proteins and classifying the consequent TFs are in a high demand.

**Results:**

The support vector machine (SVM) algorithm was utilized to construct an automatic detector for TF identification, where protein domains and functional sites were employed as feature vectors. Error-correcting output coding (ECOC) algorithm, which was originated from information and communication engineering fields, was introduced to combine with support vector machine (SVM) methodology for TF classification. The overall success rates of identification and classification achieved 88.22% and 97.83% respectively. Finally, a web site was constructed to let users access our tools (see Availability and requirements section for URL).

**Conclusion:**

The SVM method was a valid and stable means for TFs identification with protein domains and functional sites as feature vectors. Error-correcting output coding (ECOC) algorithm is a powerful method for multi-class classification problem. When combined with SVM method, it can remarkably increase the accuracy of TF classification using protein domains and functional sites as feature vectors. In addition, our work implied that ECOC algorithm may succeed in a broad range of applications in biological data mining.

## Background

Transcription factors (TFs) are special DNA-binding proteins, which are commonly recognized by RNA polymerases for transcription initiation. Under certain physiologic conditions, TFs regulate expression levels of downstream genes effectively by binding to specific DNA fragments in the promoter regions. Such a process is closely related to important biological processes such as activation of cell cycle, regulation of differentiation, and maintenance of immunologic tolerance etc [1-3]. Generally, according to their structure and function, TFs can be grouped into four classes: (1) TFs with basic domains (basic-TFs), (2) TFs with zinc-coordinating DNA binding domains (zinc-TFs), (3) TFs with Helix-turn-helix (helix-TFs), and (4) TFs with Beta-Scaffold factors (beta-TFs). It is well known that interaction mechanisms of TFs and motifs differ for different types of TFs [4-6]. Therefore, it is a momentous task to identify and classify TFs for protein functional annotation and interaction mechanism investigations in this post genome era.

Traditionally, a transcription factor, as a special case of DNA-binding protein, is identified and classified by biochemical experiments, which can be time-consuming and costly, and difficult to apply to a large scale. To overcome these defects, computational approaches are often used. Kumar et al. developed a support vector machine method to identify DNA-binding proteins[7]. Hwang et al. constructed a web server for prediction of DNA-binding residues in DNA-binding proteins, where three machine learning methods (support vector machine, kernel logistic regression and penalized logistic regression) were implemented[8]. Cho et al. built up a hidden markov model to find out possible DNA binding sites for zinc finger proteins[9]. As for transcription factors, BLAST methods were applied in most cases [10-13]. We have also constructed a simple model based on the nearest neighbor algorithm (NNA) for TF prediction in our previous work[14].

In this paper, support vector machine (SVM) and error-correcting output coding (ECOC) algorithm were utilized for TF identification and classification respectively. SVM is a method of machine learning with minimum structure risk, and it is generally employed for classification of two classes. ECOC is a method originated from information and communication engineering field, and it is commonly used to solve multi-class classification problems. Protein domains have been used as prediction signatures for protein-protein-interaction[15], protein structures[16,17], and protein sub-cellular locations[18]. On the other hand, some proteomics studies indicated close correlation exists between functional sites (such as sites of post transcriptional modification) and protein functions [19-21]. Therefore, we chose protein domains and functional sites as features to represent proteins and constructed a detector to distinguish TFs from non-TFs through a SVM method. Subsequently, a classifier based on ECOC algorithm was built to categorize TFs into four classes mentioned above. After building the detector and classifier, jackknife tests were used to assess performance of these two programs. In order to further investigate the efficiency of our approach, comprehensive comparison among BLAST, NNA, and SVM methods was carried out for TF identification, and comparison among BLAST, NNA, and ECOC was executed for TF classification. A web server was implemented to facilitate the use of these two tools.

## Results and discussion

### Identification of transcription factors

A detector was constructed based on a linear SVM model to distinguish TFs from non-TFs. We built a training data set excluding those proteins that were not annotated with any protein domains or functional sites. This training set contained 450 TFs and 1727 non-TFs [see additional file 1]. Each item of the dataset was denoted with a 4758-dimension feature vector (see "Methods" part for details).

Jackknife cross validation test was used to evaluate capability of the detector, because the jackknife cross validation test was regarded as the most objective and rigorous [22-24]. The jackknife cross validation test was operated as follows: first, for each protein in the whole training dataset, the detector was trained on the rest of the dataset (excluding the protein itself) then the trained detector was applied to predict the protein's attribute (TF or non-TF). Four measures were calculated for subsequent analysis: (1) the true positive(TP), (2) the false positive(FP), (3) the true negative(TN), (4) the false negative(FN). The true positive and the true negative were correct predictions for TFs and non-TFs respectively. A false positive occurred when a non-TF was predicted as a TF and a false negative occurred when a TF was predicted as a non-TF. Finally, the true positive rate, true negative rate, and total success rate were calculated by the following formulas:

(1) $$\left\{ \begin{array}{l} truepositiverate=\frac{TP}{TP+FN}\\ truenegativerate=\frac{TN}{TN+FP}\\ totalsuccessrate=\frac{TP+TN}{TP+FP+TN+FN} \end{array}\right.$$

Here, the "true positive rate" is the percentage of TFs predicted correctly; the "true negative rate" is the percentage of non-TFs predicted correctly; and the "total success rate" is the overall percentage of correctly predicted items (both TFs and non-TFs). Furthermore, we performed the jackknife test in several conditions, where positive and negative items were mixed in different proportions to simulate TF distribution in the natural world. The rate of positive items versus negative ones was changed from 1:1 to 1:3, with 0.5 as the step size, where the negative ones was randomly picked from the overall non-TFs datasets. SVM method was carried out for each condition in a jackknife way. Results were shown in table 1. When the numbers of positive and negative items were the same(450 versus 450), the true positive rate reached 88.44%, and the true negative rate achieved 88.00%, which meant the detector had good performance for both TF and non-TF identification. When the negative item number increased from 450 to 1350, the accuracy of the detector did not change drastically according to the true positive, true negative rate, and total success rate. Tests with different mixture rates showed that the method presented here was strong and robust.

**Table 1:** Jackknife outcomes of TF identification

**Data set size**		**Jackknife test results**		
Positive	Negative	true positive rate	true negative rate	Total success rate
450	450	88.44%	88.00%	88.22%
450	675	88.67%	89.19%	89.07%
450	900	88.44%	90.67%	89.93%
450	1125	87.56%	90.93%	89.97%
450	1350	86.67%	91.41%	90.22%

### Comparison among BLAST, NNA and SVM algorithms

In order to survey performance of the detector further, comparison among BLAST, NNA, and SVM algorithm was carried out with the dataset mentioned in paragraph of identification of transcription factor (450 positive items vs. 1727 negative items). Accuracy was calculated for positive and negative datasets respectively by the following formulas:

(2) $$\left\{ \begin{array}{l} accuracyforpositiveset=\frac{correctlypredictedpositiveitems}{totalpositiveitems}\\ accuracyfornegativeset=\frac{correctlypredictednegativeitems}{totalnegativeitems} \end{array}\right.$$

In the BLAST method, the query protein was identified as the same category as its best hit when searching similarity in the whole dataset excluding the protein itself. While in NNA method, a protein was assigned to a category with the nearest distance (see [14] for details). The distance function was defined as:

(3) $$D(x_{i},x_{k})=\frac{x_{i}.x_{k}}{\left\|x_{i}\right\|\left\|x_{k}\right\|}$$

Where, *x*_*i*_·*x*_*k *_is dot product of x_*i *_and x_*k*_, ||*x*|| is the modulus of a protein vector x. As shown in table 2, for the positive set, accuracy obtained by BLAST and NNA method was around 72% and 82%, which was lower than SVM method by about 14% and 4% respectively. While in the negative set, accuracy acquired by BLAST, NNA, and SVM method was about 74%, 93% and 91% respectively. In essential, BLAST and NNA algorithms sort an unknown item through attributes of a local item (the nearest neighbor). Hence, detectors based on these two algorithms incline to group an item into a category with a larger size. In our TF identification scenario, the number of negative set was much larger than that of the positive set, so items were more probably to be identified as negative by NNA method. Therefore the accuracy of identifying negative samples by NNA method was slightly higher than SVM algorithm. However integrated survey for both positive and negative sample indicated that SVM performed better than BLAST and NNA methods in a dataset with balanced positive and negative item numbers (data not shown). Therefore, we think performance of SVM is superior to BLAST method and comparable to the NNA method.

**Table 2:** Comparison among the BLAST, NNA, and SVM algorithm

**Total(number)**	**BLAST method**		**NNA method**		**SVM method**	
	correct number	Success rate (%)	Correct number	Success rate (%)	correct number	Success rate (%)
positive factor(450)	322	71.56	367	81.56	388	86.22
negative factor(1727)	1286	74.46	1612	93.34	1564	90.56

### Classification of transcription factors

For classification of transcription factor, the ECOC algorithm was combined with SVM method to build a multi-class classifier, which was used to categorize TFs into four classes: TFs with basic domains, TFs with zinc-coordinating DNA binding domains, TFs with Helix-turn-helix, and TFs with Beta-Scaffold factors. In our work a dataset containing 138 TFs with known class information was built. It included 37 basic-TFs, 33 zinc-TFs, 36 helix-TFs, and 32 beta-TFs [see additional file 1]. Each TF included in the dataset was presented with a 4758-dimension feature vector. Finally, in order to assess power of the multi-class classifier, the jackknife test was used to evaluate performance of both ECOC and one-against-all algorithm (one-against-all algorithm was a general algorithm for multi-class problems see "Method" part for details), in both algorithms the SVM method was employed as the basic binary classifier, and either the one-against-all or ECOC was utilized as the framework to link basic binary classifiers. The jackknife test was done as in the following: for each item in the dataset, its category was predicted using the parameters trained from the remaining items in the dataset excluding itself. Then the success rates of the four classes were calculated for the two algorithms. Equations used for success rates were given as below:

(4) $$\left\{ \begin{array}{l} Success\;rate\;for\;basic-TF=\frac{Correctly\;predicted\;basic-TF}{Totalbasic-TF\;}\\ Success\;rate\;for\;zinc-TF=\frac{Correctly\;predictedzinc-TF}{Totalzinc-TF}\\ Success\;rate\;for\;helix-TF=\frac{Correctly\;predictedhelix-TF}{Totalhelix-TF}\\ Success\;rate\;for\;beta-TF=\frac{Correctly\;predicted\;beta-TF}{Totalbeta-TF}\\ Success\;rate\;for\;overall=\frac{Correctly\;predicted\;TF}{Total\;TF} \end{array}\right.$$

Results of the one-against-all and ECOC algorithm were listed in table 3. Compared with the one-against-all algorithm, accuracy of ECOC algorithm increased notably. The success rates were improved 2.71%, 6.06%, 2.78%, and 9.37% for basic-TF, zinc-TF, helix-TF, and beta-TF respectively. For overall accuracy, the error rate was reduced from 7.25% to 2.17%. This comparison demonstrated that the ECOC method surpassed the one-against-all method for TF classification.

**Table 3:** Performance of TF classification

**Target**	one-against-all **algorithm**	**ECOC algorithm**
	Success rate	Success rate
basic-TF	35/37 = 94.59%	36/37 = 97.30%
zinc-TF	30/33 = 90.91%	32/33 = 96.97%
helix-TF	34/36 = 94.44%	35/36 = 97.22%
beta-TF	29/32 = 90.63%	32/32 = 100.00%
Overall	128/138 = 92.75%	135/138 = 97.83%

### Comparison among BLAST, NNA, and ECOC algorithms

In order to investigate performance of the multi-class classifier(depicted in paragraph of classification of transcription factor) further, comparison of BLAST, NNA, and ECOC algorithm was executed with the dataset described above (138 TFs in total, including 37 basic-TFs, 33 zinc-TFs, 36 helix-TFs, and 32 beta-TFs). BLAST and NNA methods were performed in similar ways as described in the section of comparison among BLAST, NNA, and SVM algorithms. At last, each category of TFs and total success rate was calculated for BLAST, NNA, and ECOC algorithm through formulas 4. As shown in table 4, success rates of all four TF classes were elevated to some extent when the ECOC approach was employed. Detailed analysis found that when comparing BLAST to ECOC algorithm, the maximal performance enhancement occurred in the basic-TF class, with a success rate lifted from 67.57% to 97.30%. When comparing NNA and ECOC algorithm, the biggest improvement appeared in the beta-TF class with a success rate raised from 87.50% to 100.00%. These results illuminated that ECOC method did have strong power in error correcting and fine tuning performance in multi-class categorization. When the whole dataset was considered, accuracy of BLAST and NNA was about 83% and 92%, which was around 15% and 6% lower than ECOC method respectively. This demonstrated that ECOC method outperformed greatly the BLAST and NNA methods in TF classification.

**Table 4:** Comparison among the BLAST, NNA, and ECOC algorithm

**Target**	**BLAST**	**NNA**	**ECOC**
	Success rate	Success rate	Success rate
basic-TF	25/37 = 67.57%	34/37 = 91.89%	36/37 = 97.30%
zinc-TF	29/33 = 87.88%	31/33 = 93.94%	32/33 = 96.97%
helix-TF	33/36 = 91.67%	34/36 = 94.44%	35/36 = 97.22%
beta-TF	27/32 = 84.38%	28/32 = 87.50%	32/32 = 100.00%
Overall	114/138 = 82.61%	127/138 = 92.03%	135/138 = 97.83%

### Implement

A web server for the detector and classifier has been constructed to facilitate the application of the two tools. Currently, two data types are supported by the server: Swiss-Prot AC numbers and protein sequences in FASTA format. For protein with Swiss-Prot AC numbers, information of protein domains and functional sites for the protein was extracted from the InterPro database. For a new sequence that is not covered in InterPro database, we used a program named InterProScan to screen its potential protein domains and functional sites. InterProScan is a program developed by EMBL-EBI. It combines different protein signature recognition methods into one system. Input of the program is a protein sequence with FASTA format and its output is a result file that contains InterPro entries of the sequence. Default parameters of the program were used in our research. For more detailed information of the program, please refer to webpage of InterProScan[25]. Currently, we have downloaded the program and combined it with our transcription factor tools. Users are required to provide an email address when submitting a new task. After the task is done, a reminding email will be sent to the user automatically.

## Conclusion

In this paper, an automatic detector was built for TF identification and a multi-class classifier was constructed for TF classification. Results of our work indicated that protein domains and functional sites were valid features for TF identification and classification. Moreover, our research was carried out on datasets with removed redundancy of sequence similarity, which meant our methods could provide beneficial supplement to sequence-similarity-based algorithms, such as the BLAST method, for TF identification and classification. We also believe that ECOC algorithm will have a broad application in life science, for example, classification of protein quaternary structures, categorization of kinase and prediction of protein subcellular localization etc. The detector and classifier implemented in our web server can be utilized as effective tools for TF discovery and annotation, especially for proteins with little previous knowledge. Although the two tools presented here can identify and classify TFs accurately when they have some protein domains and/or functional sites available, the two tools can not predict a protein with no protein domain or functional site annotated since this information are required in order to represent the protein in a vector. However, we believe that the impact of this limitation may become less significant since more protein domains and functional sites are obtained by biological experiments and more programs can get them directly from the protein sequences with better accuracy.

For TF identification, the SVM algorithm was employed to build the detector and performance of the detector was fairly good. Further investigations on datasets with different sample mixtures showed that the detector was robust and stable. Moreover, with protein domains and functional sites, both NNA and SVM methods perform notably better than the BLAST method. The SVM method is comparable to the NNA method for TF identification.

For TF classification, a brand-new algorithm called ECOC was introduced and employed for TF classification. In order to investigate the power of ECOC algorithm, comparison was executed in following two levels: In the first level, the ECOC algorithm was utilized as a connection framework for multi-class and was compared with a general multi-class connection algorithm named one-against-all, where the SVM method was used to build basic binary classifier for both algorithms. Comparison on this level showed that the capability of ECOC was outstanding and it surpassed the general connection algorithm for multi-class classification problems. In the second level, the ECOC was combined with SVM as the underlining method and was compared with the BLAST and NNA method. Comparison on this level indicated that the ECOC algorithm did have strong power in error correcting and fine tuning performance in multi-class categorization. Considering results of the two levels, we concluded that the ECOC combined with SVM was a powerful tool for TF classification.

## Methods

### Positive and negative datasets

In this paper, TFs and non-TFs were defined as positive and negative factors respectively. For positive factors, a primal dataset including 6464 items was extracted from TRANSFAC database v9.4[4,5]. For negative factors, the primal dataset was constructed through searching UniProt/Swiss-Prot database v10.2[26] using the following unambiguous non-TF terms: "kinase", "ubiquitin", "actin", "antigen", "biotin", "histone", "chaperon", "tubulin", "transmembrane protein", "endonuclease", "exonuclease", and "translation initiation factor". A total of 23057 entries were collected as negative factors. Subsequently, we refined the two primal datasets with the following processes: (1) filtering out proteins without Swiss-Prot accession number and those without annotation by any protein domains or functional sites, (2) eliminating redundancy in datasets against sequence similarity by program CD-HIT and PISCES with a threshold of 25% [27,28]. As a result, the final positive dataset contained 450 items, among which 138 items were with known class information; and the final negative dataset contained 1727 entries in total (Table 5).

**Table 5:** Positive and negative (TF/non-TF) datasets

**Datasets**		**Number**
TF with class information(138)	basic-TF	37
	zinc-TF	33
	helix-TF	36
	beta-TF	32
TF without class information		312
Total TF		450
Total non-TF		1727

### Feature vectors of a support vector machine

Whether a protein is a transcription factor or not is determined by its structure and function, hence it is a feasible approach to identify and classify a TF protein with protein domains and functional sites [15-21]. In this paper, we obtained information of protein domains and functional sites through InterPro database v15.0[29], which contained 14764 entries, including protein family entries, protein domain entries, and functional site entries. We noticed that there was some overlap between protein family entries and protein domain entries, or between protein family entries and functional site entries according to InterPro database documents. Therefore, we only kept those protein domains and functional sites entries. As a result, only 4758 (protein domain entry plus functional site entry) out of 14764 entries were chosen as feature vector in order to ensure vector independency. Thus features of a protein were denoted with 4758 dimension vectors. For example, if a protein X contained the 30^th ^and the 3856^th ^elements in feature list, then the 30^th ^and the 3856^th ^value were assigned to 1, the rest were set as 0. In this way, a protein can be expressed with the following equation:

(5) $$\begin{array}{ccc} X=\left[\begin{array}{l} x_{1}\\ .\\ .\\ .\\ x_{i}\\ .\\ .\\ .\\ x_{4758} \end{array}\right], & \text{where} & x_{i}=\{\begin{array}{ll} 1, & containingtheproteindomainorfunctionalsite\\ 0, & otherwise \end{array} \end{array}$$

### Support vector machine algorithm

**Figure 1:**
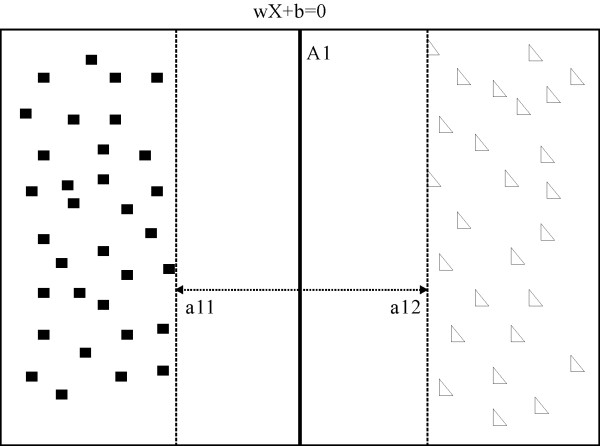
**The maximal margin hyperplane**. After sample training, the hyperplane A1 was chosen as the maximal margin hyperplane to split positive samples (black square) from negative samples (white triangle), where the maximal margin was defined as distance between a_11 _and a_12_.

The support vector machine(SVM) algorithm is based on the concept of maximal margin hyperplane which depicts the decision boundary of different categories[30,31]. In general, the hyperplane is chosen to split positive entries from negative ones with a maximal margin (figure 1). That is to say, both positive and negative categories have the greatest distances from the plane. Moreover, according to statistic learning theory, when a hyperplane has the maximal margin, it will have the highest accuracy to classify an unknown entry. The linear SVM model is an effective implementation of SVM algorithm, which builds a linear equation to depict the hyperplane through positive and negative data training. In the linear SVM model, the hyperplane can be explicitly formulated as:

(6) *w*•*X *+ *b *= 0

Where w and b are model parameters of linear SVM, and X is the feature vector of the sample. We obtained the basic SVM package from website of svmlight, which was free for academic research[32,33]. Here, when an unknown sample was represented in a feature vector of protein domains and functional sites, category Y of the sample can be predicted using the below method:

(7) $$Y=\{\begin{array}{ll} positive & \begin{array}{cc} if & w\cdot X+b>0 \end{array}\\ negative & \begin{array}{cc} if & w\cdot X+b<0 \end{array} \end{array}$$

### Error-correcting output coding algorithm

Machine learning method such as SVM is more commonly used to handle the problem of two-class. When such a method is applied to a multi-class problem, the problem should be transformed into several independent two-class tasks[34,35]. Then the method runs on each task and combines the output of these tasks. If the output of one task was wrong, the whole classifier would make incorrect classification. Error-correcting output coding algorithm (ECOC) can effectively minimize this kind of error through redundant coding information [35-37].

Considering the classification problem of TFs, there are four types of TFs which can be denoted as y_1_, y_2_, y_3_, and y_4_. In our work, one-against-all and ECOC algorithm are used to deal with the problem, where one-against-all algorithm is implemented based on previous works[38,39]. For one-against-all algorithm, 4-bit words are used to code classes. While in ECOC algorithm, the least number of bits coding m classes is 2^m-1^-l [37]. In our study, the number of classes for TFs is 4, so 7-bit words are used. For one-against-all algorithm, a class is presented as a 4-dimensions vector through naïve encoding method. While for coding in ECOC algorithm, the following rules must be taken into account so as to ensure error-correcting power of the method: (1) maximizing hamming distance for each column in encoding matrix; (2) maximizing hamming distance for each line in encoding matrix; (3) there is no complement column and line in encoding matrix. Here, in ECOC algorithm, a coding method named exhaustive codes was utilized for encoding based on previous works [36,37,40]. Detailed information of exhaustive codes was depicted as follows (while 3 ≤ m ≤ 7) [36,37,40]:

(a) For row 1, assigns ones to all bits;

(b) For row 2, consists of 2^(m-2) ^zeros followed by 2^(m-2) ^- 1 ones;

(c) For row 3, consists of 2^(m-3) ^zeros, followed by 2^(m-3) ^ones, followed by 2^(m-3) ^zeros, followed by 2^(m-3) ^- 1 ones;

(d) For row i, alternatively runs of 2^(m-i) ^zeros and ones;

According to rules mentioned above, the transformation between coding and class for one-against-all and ECOC algorithm can be visualized as in Table 6, where yes and no are mapped to 1 and 0 respectively. After encoding, four unrelated binary classifiers are built and executed independently for one-against-all algorithm. Correspondingly, seven binary classifiers are constructed for ECOC algorithm. For one-against-all algorithm, in 4-bit coding, when one binary classifier is wrong, the algorithm will make a mistake in the final results. For instance, suppose an item belongs to class y_1 _and output of four binary classifiers is 1,0,1,0. Comparing it with the 4-bit coding list, the algorithm can not correctly categorize the item because the hamming distance between the item to y_1 _and y_3 _is equal. For ECOC algorithm, in 7-bit coding, when an error occurs in an independent binary classifier, the algorithm can still properly identify the item by surplus information. For example, suppose an item belongs to class y_1 _and the output of seven binary classifiers is 1, 1, 1, 1, 1, 0, 1. Comparing it with the 7-bit coding list, we can logically draw a conclusion that the item belongs to y_1 _with maximal likelihood because the hamming distance between the item and y_1 _is the shortest. Through this mechanism, the ECOC algorithm can correct output error and improve performance of classification for multi-class problems. In our work, we established a combination classifier for TF categorization based on one-against-all and ECOC algorithms respectively, where SVM was utilized as basic classifier. Subsequently, performances of the one-against-all and ECOC algorithm were assessed by the jackknife test.

## Availability and requirements

TF website: 

**Table 6:** Coding words for multi-class task

**Class**	**one-against-all algorithm**				**ECOC algorithm**						
	Coding word				Coding word						
y1	1	0	0	0	1	1	1	1	1	1	1
y2	0	1	0	0	0	0	0	0	1	1	1
y3	0	0	1	0	0	0	1	1	0	0	1
y4	0	0	0	1	0	1	0	1	0	1	0

## Authors' contributions

GYZ developed and implemented the algorithm, collected datasets and drafted the manuscript. QLZ discussed the algorithm and collected datasets. QY implemented the algorithm. CCW and LX read and revised the manuscript. YYZ and YXL directed the whole research work and revised the manuscript. All authors read and approved the manuscript.

## Supplementary Material

Additional file 1 Swiss-Prot accession number of non-redundant training datasets. Accession number of Swiss-Prot for 450 TFs and 1727 non-TFs were included in the file. Class information for 138 TFs was also provided. Click here for file
