# Supplementary material for: The combination approach of SVM and ECOC for powerful identification and classification of transcription factor
Source: BMC Bioinformatics. 2008 Jun 16;9:282. doi: 10.1186/1471-2105-9-282 (PMC2440765; doi:10.1186/1471-2105-9-282)
Supplement: Additional file 1 — Swiss-Prot accession number of non-redundant training datasets. Accession number of Swiss-Prot for 450 TFs and 1727 non-TFs were included in the file. Class information for 138 TFs was also provided. [file 1471-2105-9-282-S1.pdf]

Swiss-Prot accession number of transcription factor(450)

O00268	O00499	O01667	O08961	O35160	O49746	O82132	P01102	P01105	P01129
P02835	P02836	P03069	P03134	P03206	P03211	P04147	P04198	P04386	P04637
P05084	P05085	P05549	P05552	P05709	P06100	P06102	P06401	P06492	P06839
P06844	P07247	P07248	P07261	P07269	P07270	P07272	P07273	P07548	P07664
P08235	P08638	P08833	P08970	P09022	P09077	P09081	P09085	P09089	P09090
P09547	P09775	P09959	P10069	P10071	P10084	P10105	P10180	P10181	P10193
P10242	P10508	P10627	P10840	P10842	P10862	P10961	P11115	P11161	P11420
P11499	P11536	P11638	P11746	P11747	P11831	P11938	P12351	P12383	P13002
P13054	P13098	P13360	P13526	P13528	P13902	P14232	P14233	P14404	P14653
P14734	P14922	P15330	P15370	P15498	P15619	P15822	P16220	P16236	P16241
P16443	P16649	P16989	P17095	P17106	P17433	P17542	P17861	P18074	P18102
P18146	P18488	P18491	P18756	P18846	P18848	P19212	P19360	P19419	P19532
P19880	P20052	P20134	P20227	P20269	P20293	P20428	P20482	P20659	P20749
P20824	P21192	P21525	P21657	P22035	P22082	P22121	P22265	P22336	P22560
P22579	P22670	P22697	P22810	P22813	P22816	P22893	P22980	P23023	P23202
P23255	P23615	P23683	P23760	P23769	P23803	P23906	P24278	P24345	P24349
P25032	P25042	P25302	P25364	P25425	P25799	P25932	P25991	P26017	P26343
P26359	P26370	P26754	P26798	P26801	P27347	P27540	P27692	P27705	P27889
P27898	P28159	P28167	P28324	P28362	P28574	P29056	P29084	P29303	P29454
P29747	P31260	P31263	P31264	P31273	P31314	P31367	P31384	P32183	P32333
P32338	P32389	P32445	P32479	P32480	P32558	P32591	P32607	P32773	P32774
P32776	P32780	P32805	P32862	P33076	P33244	P33308	P33339	P33400	P33748
P34228	P34233	P34707	P34708	P34765	P34909	P35189	P35428	P35452	P35632
P35638	P35710	P35817	P35869	P36011	P36100	P36130	P36395	P36611	P36622
P36627	P36631	P36956	P37366	P37826	P38114	P38128	P38141	P38165	P38530
P38699	P38704	P38717	P38749	P38806	P38827	P38830	P38889	P38915	P39001
P39008	P39073	P39113	P39521	P39678	P39720	P39880	P40068	P40209	P40349
P40427	P40469	P40535	P40573	P40578	P40798	P40956	P40971	P41157	P41164
P41182	P41546	P41696	P41813	P41895	P41896	P41933	P41934	P42230	P42232
P42282	P43345	P43569	P43680	P46581	P46676	P46677	P46678	P46692	P46954
P46974	P46995	P47175	P47821	P47977	P47988	P48361	P48985	P49640	P49698
P49848	P49866	P49906	P50104	P50105	P50220	P50575	P50947	P51123	P51179
P51558	P51843	P51948	P51974	P52654	P52890	P53032	P53040	P53050	P53064
P53094	P53438	P53566	P53743	P53968	P54000	P54785	P54845	P55316	P56915
P78411	P91943	P93835	P93839	P97503	Q00420	Q00453	Q00578	Q00916	Q00969
Q00978	Q01094	Q01159	Q01371	Q01522	Q01664	Q01714	Q01822	Q01842	Q01981
Q02100	Q02206	Q02283	Q02336	Q02457	Q02486	Q02556	Q02638	Q02983	Q03017
Q03062	Q03123	Q03125	Q03164	Q03465	Q03654	Q04226	Q04635	Q04650	Q04667
Q04673	Q04688	Q05021	Q05066	Q05192	Q05344	Q05466	Q05738	Q05913	Q05950
Q05959	Q06433	Q06596	Q06706	Q06945	Q07472	Q07687	Q08050	Q12030	Q12041
Q12206	Q12514	Q13952	Q14119	Q14872	Q15543	Q15697	Q15853	Q17381	Q17396
Q24142	Q24143	Q24206	Q24266	Q24338	Q26635	Q38895	Q39028	Q39155	Q39235
Q39260	Q40476	Q40477	Q40478	Q40479	Q42575	Q43614	Q60542	Q60636	Q60688
Q60793	Q60987	Q61026	Q61164	Q61575	Q61985	Q62722	Q63046	Q63187	Q63244
Q63753	Q64732	Q91839	Q91853	Q92731	Q98875	Q99814	Q9UH73	Q9Z205	Q9ZWM9

Swiss-Prot accession number of non transcription factor(1727)

A0JN69	A1X157	A2VDL8	O00311	O00399	O00443	O00444	O00459	O00499	O01510
O01991	O02697	O02703	O02810	O05543	O05871	O06079	O06081	O07086	O07898
O07899	O07900	O08788	O08874	O13731	O13733	O13817	O13839	O13889	O13932
O14328	O14350	O14523	O14525	O14618	O14936	O14976	O15021	O15818	O16277
O18209	O22040	O23372	O24585	O26929	O27995	O28739	O29470	O29666	O29910
O31540	O31663	O32153	O32156	O32221	O32848	O34368	O34638	O34664	O35094
O35099	O35569	O35600	O35926	O41933	O42282	O42632	O42894	O42900	O43066
O43077	O43155	O43164	O43283	O43422	O43572	O43586	O43683	O43818	O44406
O44739	O44757	O46560	O46607	O48850	O49203	O49227	O52655	O52728	O54101
O54709	O54830	O54991	O55034	O58969	O59739	O59836	O60017	O60079	O60125
O60264	O60291	O60337	O60524	O60739	O60763	O60841	O60844	O60870	O61661
O63264	O64474	O67174	O67879	O68562	O70405	O73777	O73943	O74208	O74248
O74442	O74526	O74539	O74630	O74815	O74925	O75146	O75154	O75391	O75409
O75460	O75494	O75569	O75602	O76039	O77617	O78474	O80536	O80568	O83433
O84121	O84417	O84419	O84462	O84880	O86230	O87120	O87394	O87656	O88627
O88875	O93654	O94269	O94324	O94513	O94603	O94647	O94751	O94953	O95071
O95202	O95487	O95711	O95835	P00522	P00536	P00545	P00557	P00996	P01831
P01882	P02829	P03073	P03076	P03153	P03177	P03179	P03182	P03218	P03243
P03701	P03880	P03949	P03987	P04072	P04185	P04233	P04294	P04385	P04413
P05725	P05825	P06501	P06729	P06916	P07072	P07074	P07211	P07277	P07527

P08018	P08571	P08575	P08922	P09319	P09326	P09564	P09888	P09932	P09979
P0A0V2	P0A1I2	P0A1K7	P0A1K9	P0A234	P0A3U4	P0A4M7	P0A4N1	P0A5B8	P0A5N9
P0A5Z9	P0A619	P0A647	P0AA48	P0AA65	P0AA69	P0AA74	P0AA94	P0AAE1	P0ABT9
P0AD04	P0AD29	P0AD47	P0ADJ8	P0ADP6	P0AE35	P0AE47	P0AEB6	P0AEC2	P0AEC7
P0AEI0	P0AEL5	P0AEW8	P0AF81	P0AFN8	P0AFQ0	P0AFW6	P0C0L8	P0C0T1	P0C198
P0C1B1	P0C219	P0C264	P0C536	P10047	P11049	P11075	P11108	P11461	P11792
P11912	P12136	P12401	P12812	P12868	P13036	P13186	P13227	P13288	P13299
P13329	P13340	P13423	P13632	P13651	P13765	P13779	P13830	P13895	P14202
P14285	P14376	P14422	P14432	P14680	P15287	P15379	P15442	P15443	P15711
P15921	P16010	P16405	P16452	P16462	P16497	P16563	P16789	P16945	P17261
P17423	P18009	P18060	P18091	P18106	P18150	P18160	P18294	P18431	P18475
P18622	P18790	P19256	P19361	P19390	P19525	P19617	P19680	P19807	P19812
P19829	P19895	P20007	P20020	P20037	P20162	P20485	P20690	P20736	P20794
P21077	P21250	P21268	P21293	P21505	P21778	P21849	P21854	P21860	P21893
P21919	P21978	P22006	P22040	P22080	P22081	P22216	P22804	P23292	P23354
P23497	P23504	P23525	P23561	P23588	P23647	P23735	P24005	P24081	P24521
P24587	P24719	P24814	P24852	P25055	P25332	P25333	P25359	P25360	P25386
P25574	P25848	P26266	P26362	P26401	P26403	P26842	P26948	P27206	P27426
P27613	P27801	P28067	P28068	P28284	P28627	P28707	P28708	P28722	P28791
P28925	P28966	P28990	P29228	P29481	P29725	P29939	P30149	P30203	P30291
P30344	P30636	P30847	P31064	P31106	P31374	P31492	P31631	P32011	P32072
P32225	P32264	P32350	P32361	P32368	P32481	P32562	P32581	P32600	P32678
P32742	P32761	P32790	P32791	P32801	P32839	P32866	P32944	P33015	P33020
P33116	P33279	P33288	P33294	P33698	P33802	P33941	P33973	P34101	P34102
P34103	P34104	P34125	P34152	P34167	P34206	P34244	P34314	P34333	P34478
P34544	P34552	P34576	P34607	P34635	P34649	P34749	P34756	P34892	P34894
P35114	P35141	P35202	P35546	P35578	P35590	P35597	P35762	P35790	P35865
P35983	P36003	P36028	P36029	P36096	P36232	P36497	P36615	P37034	P37142
P37297	P37349	P37433	P37511	P37562	P37608	P37624	P37642	P37661	P37665
P37669	P37733	P37739	P37740	P37908	P38008	P38080	P38110	P38147	P38262
P38292	P38323	P38324	P38329	P38360	P38369	P38623	P38691	P38692	P38792
P38912	P38943	P38970	P38990	P38993	P39073	P39075	P39104	P39241	P39277
P39282	P39288	P39314	P39325	P39542	P39830	P39838	P39842	P39928	P39986
P40034	P40036	P40075	P40107	P40217	P40259	P40318	P40358	P40433	P40438
P40445	P40530	P40548	P40758	P40876	P40985	P41060	P41217	P41218	P41279
P41374	P41484	P41720	P41888	P41949	P42071	P42159	P42243	P42245	P42292
P42356	P42411	P42424	P42527	P42951	P43109	P43403	P43565	P43568	P43633
P43682	P44250	P44482	P44543	P44622	P44833	P45249	P45288	P45394	P45594
P45951	P45996	P46026	P46106	P46136	P46139	P46224	P46560	P46599	P46614
P46680	P46730	P46920	P46962	P47042	P47068	P47104	P47116	P47260	P47311
P47391	P47458	P47545	P48240	P48479	P48553	P48960	P48988	P49025	P49321
P49594	P49605	P49657	P49762	P49982	P50053	P50186	P50443	P50541	P50582
P50636	P51145	P51321	P51392	P51813	P51950	P51957	P52047	P52101	P52143
P52446	P52484	P52560	P52569	P52636	P52824	P53009	P53039	P53063	P53109
P53119	P53153	P53170	P53193	P53233	P53235	P53273	P53281	P53331	P53394
P53599	P53739	P53746	P53801	P53838	P53904	P53925	P54190	P54199	P54211
P54301	P54302	P54352	P54674	P54677	P54734	P54736	P54738	P54742	P54883
P55183	P55471	P55568	P55669	P55734	P55824	P55827	P55829	P55835	P55839
P55884	P55980	P56287	P56288	P56848	P56975	P57034	P57041	P57058	P57078
P57217	P57368	P57993	P58216	P58593	P58754	P59025	P59114	P59963	P60008
P61170	P61325	P61584	P61916	P63358	P63391	P63399	P63770	P64144	P65007
P65371	P65733	P65930	P67117	P67730	P68746	P69525	P70715	P70718	P70954
P71356	P72732	P73009	P73450	P73515	P73771	P74297	P74436	P75109	P75205
P75264	P75444	P75516	P75524	P75551	P75613	P75785	P75835	P75955	P76014
P76016	P76042	P76219	P76221	P76236	P76335	P76339	P76419	P77172	P77269
P77400	P77493	P77504	P77510	P77536	P77599	P77866	P78395	P79280	P80197
P80444	P80659	P81344	P81346	P81958	P82602	P83075	P83097	P83102	P83103
P83736	P83977	P83985	P84731	P87050	P87295	P88963	P90495	P91133	P91167
P91928	P94799	P96661	P96680	P97364	P97793	P98153	P98160	Q00094	Q00095
Q00366	Q00497	Q00537	Q00595	Q00609	Q00964	Q01000	Q01013	Q01151	Q01389
Q01397	Q01583	Q01590	Q01621	Q01969	Q02073	Q02099	Q02192	Q02595	Q02725
Q02907	Q02937	Q02938	Q02952	Q03002	Q03016	Q03043	Q03102	Q03193	Q03215
Q03228	Q03264	Q03306	Q03435	Q03533	Q03563	Q03650	Q03697	Q03702	Q03730
Q03778	Q03877	Q04216	Q04432	Q04491	Q04594	Q04637	Q04641	Q04800	Q04835
Q04839	Q04956	Q05084	Q05181	Q05439	Q05609	Q05652	Q05672	Q05999	Q06067
Q06098	Q06147	Q06169	Q06328	Q06648	Q06651	Q06850	Q06904	Q07071	Q07108
Q07292	Q07408	Q07444	Q07457	Q07617	Q07622	Q07835	Q07837	Q07929	Q07963
Q08017	Q08109	Q08213	Q08224	Q08430	Q08444	Q08722	Q08732	Q08794	Q08853

Q08D99	Q08DV0	Q09092	Q09103	Q09298	Q09349	Q09427	Q09488	Q09573	Q09629
Q09639	Q09690	Q09722	Q09738	Q09766	Q09809	Q09879	Q09898	Q09931	Q0H8X2
Q0H8X7	Q0H8X8	Q0H8X9	Q0H8Y3	Q0H8Z3	Q0P496	Q0P4K8	Q0P5H8	Q0V8S0	Q0VCA9
Q0VCB1	Q0VCR6	Q0VD34	Q0VD42	Q0WNY5	Q10124	Q10156	Q10169	Q10242	Q10357
Q10435	Q10447	Q10475	Q10699	Q10875	Q10900	Q10925	Q11076	Q11090	Q12003
Q12149	Q12220	Q12236	Q12263	Q12265	Q12277	Q12298	Q12310	Q12333	Q12373
Q12449	Q12469	Q12471	Q12505	Q12674	Q12675	Q12697	Q12745	Q12802	Q12866
Q12929	Q13023	Q13099	Q13164	Q13191	Q13233	Q13308	Q13309	Q13323	Q13470
Q13523	Q13546	Q13554	Q14004	Q14008	Q14152	Q14247	Q14296	Q14669	Q149N8
Q14CZ7	Q15075	Q15118	Q15643	Q15645	Q15652	Q15750	Q15762	Q15814	Q16667
Q16943	Q17632	Q19192	Q19238	Q19266	Q1BYA7	Q1JPH6	Q1KKW7	Q1RJB3	Q1S9I9
Q21735	Q22258	Q22366	Q22647	Q22695	Q23977	Q24145	Q24572	Q24592	Q24740
Q25479	Q26539	Q26768	Q27178	Q27324	Q27489	Q28205	Q28433	Q29RU0	Q2EHL8
Q2FED6	Q2HJA8	Q2J862	Q2JJ30	Q2KIU0	Q2KJF7	Q2LGB3	Q2QTC2	Q2T9U5	Q2U9B0
Q32KS0	Q32L68	Q32L97	Q32LN5	Q32MK0	Q38419	Q38SD2	Q3IEY8	Q3J6C1	Q3L8P3
Q3LRP3	Q3SWZ4	Q3SX26	Q3SY52	Q3SZI1	Q3SZJ2	Q3SZM9	Q3T133	Q3U145	Q3UF64
Q3UGM2	Q3UV71	Q3V129	Q3ZBG5	Q3ZC12	Q3ZCF3	Q40545	Q42736	Q44636	Q44848
Q46036	Q46L57	Q47068	Q47162	Q47377	Q49396	Q49430	Q496M5	Q49MI3	Q49VU5
Q4A0G5	Q4IK03	Q4JU24	Q4P0K0	Q4PH16	Q4R5B8	Q4V7X9	Q4VBH4	Q4VSN4	Q4WHB7
Q502L7	Q504Y2	Q50585	Q50862	Q50863	Q50864	Q52107	Q52664	Q52665	Q52969
Q53R41	Q55774	Q55NZ6	Q561M0	Q56646	Q56902	Q56JZ5	Q56UN5	Q57190	Q571I4
Q571K4	Q57242	Q57519	Q57710	Q57PI7	Q57QC4	Q58030	Q58129	Q58387	Q58957
Q59014	Q59081	Q59263	Q59H18	Q5AGC7	Q5B367	Q5B8L2	Q5E999	Q5E9D0	Q5EA99
Q5EAB0	Q5EAK6	Q5F349	Q5F361	Q5F3W3	Q5F486	Q5GLZ8	Q5HEI2	Q5K4L6	Q5KBI0
Q5KFE0	Q5KJQ4	Q5KSL6	Q5M775	Q5PH63	Q5QJ74	Q5R9A7	Q5R9W2	Q5REG1	Q5RKV6
Q5S007	Q5S7T7	Q5T0T0	Q5T3F8	Q5T447	Q5TCY1	Q5U5R9	Q5U651	Q5UNT7	Q5UNZ1
Q5UPT4	Q5UPU3	Q5UPW7	Q5UPY4	Q5UPZ2	Q5UQ57	Q5UQ77	Q5UQ88	Q5UQ94	Q5UQC1
Q5UQJ6	Q5UQM4	Q5UQR3	Q5UQW7	Q5UR39	Q5UR48	Q5VK71	Q5VT25	Q5VU65	Q5VWQ8
Q5WL39	Q5XPI4	Q5Z6B1	Q5Z987	Q5ZJB4	Q5ZJS6	Q5ZLP2	Q5ZPR3	Q60030	Q60053
Q60337	Q60739	Q60767	Q61476	Q61K76	Q62101	Q62137	Q621J7	Q62388	Q62407
Q62472	Q62IJ6	Q63111	Q63572	Q63994	Q63A51	Q641K5	Q66T72	Q6ARA9	Q6AYH3
Q6AZD4	Q6BH37	Q6BLM3	Q6BV76	Q6CAD2	Q6CFE7	Q6CNN5	Q6CSA1	Q6D8D5	Q6DCL5
Q6DH44	Q6F1S1	Q6FNV5	Q6FX42	Q6FY22	Q6G0G3	Q6GED5	Q6IQ26	Q6J9G1	Q6L5D4
Q6MAN0	Q6NJ43	Q6NYK8	Q6P4S6	Q6PAQ4	Q6PEI3	Q6PGG2	Q6Q0C0	Q6RYW5	Q6STE5
Q6TEM9	Q6UX27	Q6UXF1	Q6UXZ0	Q6UY09	Q6WKZ4	Q6XE24	Q6YHK3	Q6YXY2	Q6ZMT4
Q6ZNA4	Q70EK8	Q70FG9	Q70PP2	Q71H61	Q754T3	Q756G2	Q75CC8	Q75CQ8	Q75D46
Q75LI2	Q76E23	Q76N89	Q7AJA5	Q7BCK4	Q7JVI3	Q7KRY6	Q7L622	Q7L8L6	Q7MV19
Q7RZT9	Q7T0B0	Q7T6X2	Q7TMI3	Q7TMJ8	Q7TMY8	Q7TT18	Q7TXA9	Q7UFZ2	Q7VL53
Q7VNT5	Q7WR85	Q7XRU4	Q7YU24	Q7Z2X4	Q7Z2Y5	Q7Z419	Q7Z465	Q7Z692	Q7Z695
Q7ZW16	Q7ZYJ3	Q80V03	Q80XG9	Q80YE7	Q80ZI6	Q82AG6	Q83IA0	Q84JU6	Q84V18
Q84Y18	Q86MW9	Q86TB3	Q86UR5	Q86UX6	Q86WB0	Q86WC6	Q86XD8	Q86Y13	Q86YD3
Q874C1	Q893Q9	Q89A17	Q89AD1	Q8AXY6	Q8AYG3	Q8BG60	Q8BGN6	Q8BH01	Q8BKX6
Q8BLK3	Q8BMZ5	Q8BVE8	Q8BX57	Q8BY79	Q8BZT2	Q8CEE6	Q8CGF5	Q8CI61	Q8CJ53
Q8G4G1	Q8G5X4	Q8G838	Q8GGL1	Q8GUQ5	Q8GY23	Q8HSW1	Q8HVK8	Q8HY03	Q8IR79
Q8ISF8	Q8IU80	Q8IV63	Q8IVH8	Q8IW41	Q8IWF7	Q8IWL3	Q8IWU2	Q8IX05	Q8IZ96
Q8IZR5	Q8IZV2	Q8JFV8	Q8JZW8	Q8K1R7	Q8K450	Q8KA87	Q8KAW7	Q8KG79	Q8L4H4
Q8L5Y9	Q8L718	Q8L850	Q8LBB2	Q8LBL5	Q8LE94	Q8LJT8	Q8LPT9	Q8MR31	Q8MT36
Q8MYQ1	Q8N0W3	Q8N1G1	Q8N3G9	Q8N568	Q8NA54	Q8NA82	Q8NBS3	Q8NCE0	Q8NE28
Q8NG66	Q8NHG8	Q8PK29	Q8PNH3	Q8PTT8	Q8PZ69	Q8PZI1	Q8R550	Q8R5A3	Q8R5N8
Q8RQL4	Q8RY67	Q8S403	Q8T0S6	Q8TAD8	Q8TAG5	Q8TAQ2	Q8TAS1	Q8TAZ6	Q8TBB1
Q8TBE7	Q8TDC3	Q8TDZ2	Q8TE04	Q8TF76	Q8TGY9	Q8TK65	Q8TR06	Q8TZB3	Q8VC56
Q8VDX6	Q8VHF0	Q8VIG6	Q8VL02	Q8VY05	Q8VZ40	Q8W2F3	Q8WUG5	Q8WUJ0	Q8WWG1
Q8WWV3	Q8WXI2	Q8X1E6	Q8X5R9	Q8X6R9	Q8X6V3	Q8X8G6	Q8XBV9	Q8XJL8	Q8XN03
Q8XU11	Q8XX83	Q8Y0M0	Q8YD73	Q8Z6P7	Q8ZBM0	Q91618	Q91VE6	Q91VK4	Q91ZU6
Q920P5	Q92212	Q92213	Q92266	Q92667	Q926C3	Q92JG1	Q92JP8	Q92ZT0	Q93IE8
Q93Z13	Q93Z66	Q94125	Q94AU2	Q95050	Q95L46	Q95MP0	Q95Q98	Q95SP2	Q95UN8
Q95YI5	Q95ZQ4	Q969G3	Q969I6	Q96A29	Q96BF3	Q96BH1	Q96D53	Q96DZ9	Q96EP1
Q96GX5	Q96JH7	Q96L96	Q96LW7	Q96M32	Q96NA2	Q96PY6	Q96QF0	Q96QP1	Q96QV1
Q96QZ7	Q96RR4	Q96RY7	Q96S38	Q96T58	Q97BQ3	Q97P44	Q98I54	Q98QW4	Q98ST5
Q99189	Q99385	Q99435	Q99467	Q99558	Q99570	Q99759	Q99795	Q99942	Q99996
Q99J86	Q99L47	Q99LJ5	Q99MQ3	Q99P91	Q9AL99	Q9ASX5	Q9BQA5	Q9BRS2	Q9BTE1
Q9BVS4	Q9BX93	Q9BXU1	Q9BY77	Q9BYE2	Q9BYK8	Q9BYT3	Q9BZD7	Q9BZF9	Q9BZM2
Q9C098	Q9C0K7	Q9CGE3	Q9CNP2	Q9CWK3	Q9CZX5	Q9D0K1	Q9D2Y4	Q9D733	Q9D8T7
Q9DAS4	Q9DBH5	Q9DCJ7	Q9DE14	Q9DEI1	Q9EPQ2	Q9ERA6	Q9ERE9	Q9ESL4	Q9ESN9
Q9EVN3	Q9EXQ1	Q9FAB3	Q9FIY7	Q9FY46	Q9GKI7	Q9GKZ4	Q9H074	Q9H0C8	Q9H0E2
Q9H295	Q9H2D6	Q9H2G2	Q9H2Y9	Q9H422	Q9H479	Q9H4A3	Q9H4L7	Q9H6Y7	Q9H792
Q9H7Z6	Q9H9L3	Q9HB10	Q9HBA9	Q9HBG6	Q9HBG7	Q9HBL6	Q9HCI5	Q9HCK5	Q9HCN3
Q9HCU5	Q9HDC9	Q9HFF4	Q9HG09	Q9HKY3	Q9HNN8	Q9HTX3	Q9I7F7	Q9J5B1	Q9JHR9

Q9JI90 Q9JLN5 Q9JLT6 Q9JLV1 Q9JLV6 Q9JM01 Q9JRP9 Q9KFF1 Q9KPI5 Q9KVI9
Q9KWM8 Q9L473 Q9LKI5 Q9LN71 Q9LNE4 Q9LRB7 Q9LX30 Q9LYU8 Q9M081 Q9M3G7
Q9M3H5 Q9MA15 Q9NB71 Q9NH13 Q9NI63 Q9NIV1 Q9NLA1 Q9NPC3 Q9NPF0 Q9NPJ1
Q9NQC7 Q9NR48 Q9NRA0 Q9NRH2 Q9NRP7 Q9NS56 Q9NS61 Q9NSE7 Q9NSU2 Q9NSY1
Q9NW38 Q9NWF9 Q9NYB0 Q9NYY8 Q9NZ20 Q9NZC9 Q9P0M2 Q9P0M6 Q9P286 Q9P2D0
Q9P2H3 Q9P3U0 Q9P4Z1 Q9P6Y2 Q9P7Q7 Q9P7S5 Q9P7V9 Q9P7X5 Q9PIN2 Q9PJ54
Q9PJH1 Q9PK27 Q9PL45 Q9PPN9 Q9PQV8 Q9QUN5 Q9QUR7 Q9QXK2 Q9QXL8 Q9QY17
Q9QZ05 Q9R0A0 Q9R1A8 Q9R1N3 Q9RI12 Q9RUE8 Q9S3R9 Q9SCY5 Q9SPL2 Q9T074
Q9T0G7 Q9TT99 Q9TV52 Q9TVW2 Q9TY95 Q9U1Y5 Q9U308 Q9U8G7 Q9UBL3 Q9UGJ0
Q9UGN4 Q9UHB6 Q9UHD2 Q9UHI5 Q9UHK0 Q9UHN6 Q9UKG1 Q9UL54 Q9ULT8 Q9ULU4
Q9ULV3 Q9UR66 Q9US59 Q9UST1 Q9UTH3 Q9V3I5 Q9V3Q6 Q9V4L4 Q9V853 Q9V8P9
Q9VB08 Q9VBW3 Q9VEZ5 Q9VH48 Q9VHH9 Q9VN14 Q9VPC0 Q9VR91 Q9VRP9 Q9VRX3
Q9VUX2 Q9VW97 Q9VXG8 Q9VXK6 Q9VXY2 Q9VYD1 Q9W0K0 Q9W0S9 Q9W0V1 Q9W391
Q9WTJ4 Q9WX70 Q9XHM1 Q9XJJ7 Q9XT50 Q9Y237 Q9Y2A5 Q9Y2B0 Q9Y2I7 Q9Y2S7
Q9Y2U8 Q9Y3A4 Q9Y3A6 Q9Y3R0 Q9Y484 Q9Y4P8 Q9Y572 Q9Y616 Q9Y6J8 Q9Y6R4
Q9Y6Y8 Q9Y7W4 Q9YAW0 Q9YC05 Q9YDD2 Q9YDY8 Q9YG68 Q9Z172 Q9Z1S0 Q9Z1W9
Q9Z1Z2 Q9Z3D6 Q9Z3Q5 Q9Z6U5 Q9ZCI7 Q9ZHD4 Q9ZZX0

Classification information of 138 transcription factor
TF with Basic domains(37)
P04198 P11115 P14232 P20824 Q04667 P19532 P11420 P21525 P13098 P16443
P22816 P41546 P52890 P09775 P18846 P25032 Q01664 P13526 P20482 P54845
P22560 P25302 P03206 P13902 P17861 P16220 P22697 P14233 P35869 P01102
P19880 P26798 Q15853 P18491 P29303 P36622 P46581
TF with Beta-Scaffold factors(32)
P35710 Q40479 O82132 P13002 P16236 P25799 Q13952 P04637 P36611 P43680
Q05066 P20227 P27347 Q03017 Q63046 P10840 Q40476 P11831 P20749 P35632
P42232 P93835 Q40478 Q07472 Q05344 P11746 P15330 P25042 Q05738 P42230
Q40477 Q06945
TF with Helix-turn-helix(36)
P09077 P28167 P97503 P10181 P22810 P31367 P39880 Q02283 P09085 P18488
P24345 P28362 Q01094 Q60987 P01105 P07269 P14734 P22121 P29454 P46692
Q05466 P02836 P07548 P17433 P23683 P41934 P05552 P31260 P49640 Q01822
Q05950 P10961 P27898 Q04650 P10180 P19419
TF with Zinc-coordinating DNA binding domains(33)
P18102 P23803 P42282 P07247 P51179 P14404 P10508 P32338 Q24142 P05085
P11638 P36627 P27705 P34708 Q03164 P10071 P15370 P19212 P37826 P08638
P23769 P06401 P12351 P39001 Q01981 P04386 P11161 P18146 P21192 Q60793
P07248 P13054 P51843
